# The association between constipation and subsequent risk of atopic dermatitis in children: the Japan Environment and Children’s Study

**DOI:** 10.1265/ehpm.23-00103

**Published:** 2023-11-16

**Authors:** Yoshihiko Takano, Yuri Aochi, Satoyo Ikehara, Kanami Tanigawa, Sachiko Baba, Keiichi Ozono, Tomotaka Sobue, Hiroyasu Iso

**Affiliations:** 1 Division of Environmental Medicine and Population Sciences, Department of Social and Environmental Medicine, Graduate School of Medicine, Osaka University, 2-2 Yamadaoka Suita City, Osaka 565-0871, Japan; 2 Department of Pediatrics, Sakai City Medical Center, 1-1-1 Ebaraji-cho, Nishi-ku, Sakai City, Osaka 593-8304, Japan; 3 Osaka Regional Center for Japan Environment and Children’s Study (JECS), Osaka University, 1-3 Yamadaoka Suita City, Osaka 565-0871, Japan; 4 Osaka Maternal and Child Health Information Center, Osaka Women’s and Children’s Hospital, 840 Murodo-cho, Izumi City, Osaka 594-1101, Japan; 5 Department of Pediatrics, Osaka University Graduate School of Medicine, 2-2 Yamadaoka, Suita City, Osaka 565-0871, Japan; 6 Institute for Global Health Policy Research, Bureau of International Health Cooperation, National Center for Global Health and Medicine, 1-21-1 Toyama, Shinjuku-ku, Tokyo 162-8655, Japan

**Keywords:** Atopic dermatitis, Child, Cohort study, Constipation, Dysbiosis, Eczema, Gut microbiota

## Abstract

**Background:**

No study has examined the association between constipation and atopic dermatitis (AD) in infants and toddlers. We aimed to explore that association in toddlers using the data from a nationwide birth cohort study.

**Methods:**

From the Japan Environment and Children’s Study, a nationwide prospective birth cohort study that began in 2011, children born in a singleton live birth were analyzed. Participants completed questionnaires containing questions related to bowel movements and AD, during 1.5 to 3 years after birth. Constipation at 1 year of age was defined as having ≤2 bowel movements per week. AD was defined based on participant’s responses to the modified ISAAC questionnaire and/or self-reported physician’s diagnosis. Outcome was defined as the cumulative number of AD cases that occurred until 3 years of age. Adjusted odds ratios (ORs) and 95% confidence intervals (CIs) for development of AD were calculated by a multivariable logistic regression.

**Results:**

From a total of 62,777 participants who met the study inclusion criteria, 14,188 children (22.6%) were affected by AD between the ages of 1.5 and 3 years. The adjusted OR of developing AD for the presence versus absence of constipation at 1 year of age was 1.18 (95% CI, 1.01–1.38).

**Conclusion:**

Constipation at 1 year of age was associated with a slightly higher risk of AD until 3 years of age.

**Supplementary information:**

The online version contains supplementary material available at https://doi.org/10.1265/ehpm.23-00103.

## Introduction

Atopic dermatitis/eczema (AD) is a skin disease commonly seen around the world, affecting approximately 10% to 20% of children in Japan [1–3]. Although the pathogenesis of AD has not been fully elucidated, multiple genetic and environmental factors are thought to influence the onset and progress of the disease [1, 4, 5]. With the recent improvements in genetic analysis technology, comprehensive genetic studies of the gut microbiota revealed that the diversity of the gut microbiota was reduced in children with AD, called as dysbiosis [6–8].

As for functional constipation defined as constipation with no underlying organic cause, studies of stools in children with constipation have shown that dysbiosis affects constipation through a variety of mechanisms including, (1) changes in the composition of the intestinal microflora, such as an increase in the ratio of Clostridium spp. and Bifidobacterium spp., (2) changes in peristaltic movement due to the action of gases and organic substances produced by the metabolism of the intestinal microflora, (3) changes in peristalsis due to neuroendocrine factors such as gastrin, serotonin, and motilin [9–11]. Previously two epidemiological studies of adolescents and adults examined the association between constipation and AD [12, 13]; both of which reported that constipation was a risk factor for the development of AD. However, no such study has examined this issue in children, especially in infants and toddlers.

In the present study, we aimed to investigate whether functional constipation at 1 year of age is a risk factor for the development of AD.

## Methods

### Study design

The present study analyzed data from the Japan Environment and Children’s Study (JECS), a government-funded, prospective birth cohort study that began in January 2011 [14, 15]. For the JECS, 15 Regional Centres were selected: Hokkaido, Miyagi, Fukushima, Chiba, Kanagawa, Koshin, Toyama, Aichi, Kyoto, Osaka, Hyogo, Tottori, Kochi, Fukuoka and Southern Kyushu/Okinawa. Expectant women who were living in the Study Area at the time of enrollment; who were expected to continue to live there for the foreseeable future; whose due date of delivery was between January 2011 and March 2014; and who were able to participate in the study without difficulty (that is, had adequate Japanese language comprehension to completely respond to self-administered questionnaire), were included in the JECS study population. Details of the JECS protocol have been described elsewhere [14, 15]. We obtained outcomes, exposures, and covariates data through self-administered questionnaires, and medical record transcriptions. In JECS, the questionnaires were distributed at enrollment, during the second or third trimesters of pregnancy, one month after childbirth, and every six months after childbirth until 3 years of age. In addition, medical records, at enrollment, delivery, and one month after birth, were transcribed by physicians, midwives/nurses, and/or Research Co-ordinators.

### Study population

**Fig. 1:**
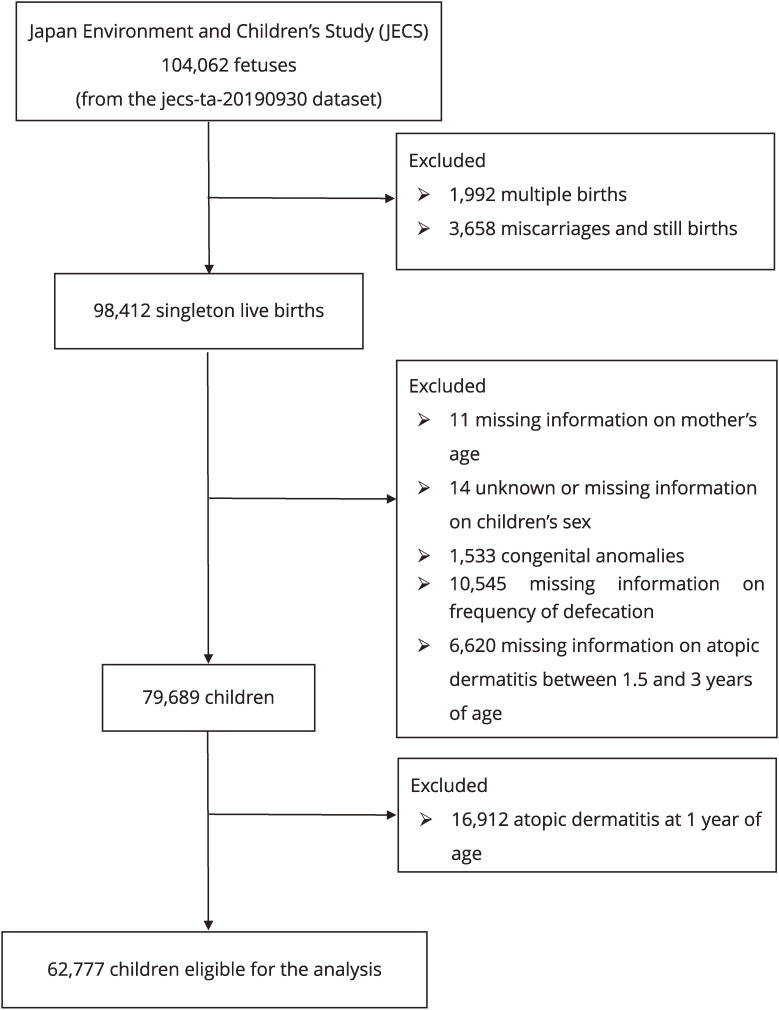
Flow chart of the participant selection

We used the jecs-ta-20190930 data set, which was released in October 2019 and revised in June 2021. Among 104,062 fetal records, 98,412 singleton live births were included. Following cases were excluded: missing information on mother’s age (n = 11), unknown or missing information on children’s sex (n = 14), congenital anomalies which potentially cause constipation (n = 1,533), missing information on frequency of defecation (n = 10,545), and missing information on AD (n = 6,620). The missing information on AD was defined as no response to the question on AD at 1.5, 2, and 3 years of age. Also, children with the presence of AD at 1 year of age were excluded (n = 16,912). The final population of this study comprised of 62,777 participants (Fig. 1).

### Exposure variables

From the questionnaire, we obtained data regarding constipation at 1 year of age. Constipation in young children is mostly functional constipation [16], which is often diagnosed by the ROME criteria when, two or more of the six questions in the questionnaire are satisfactorily answered [17, 18]. In the JECS questionnaire, there are only two sequential questions on constipation for the age of 1 year of age, “does your child poop almost every day?”, and “if not, how many times a week does your child poop?”. Based on the response, we defined “constipated” as having ≤2 bowel movements per week, and “normal” as defecation almost every day, which we used as a reference group.

### Outcome measures

We obtained the data regarding AD from the questionnaires at 1.5, 2, and 3 years of age. Outcome was defined as the cumulative number of AD cases that occurred between 1.5 and 3 years of age, and the number of cases were counted if AD occurred at least once in the questionnaire at 1.5, 2, or 3 years of age. The definition of AD was based on the answer of “yes” to the question, “Has your child ever had a recurring itchy rash for at least two months? (in the questionnaire at 1.5 years of age)”, and “Has your child ever had an itchy recurring rash for at least 6 months? (in the questionnaires at 2 and 3 years of age)”. These questions are partly adapted from Japanese version of the ISAAC questionnaire for ages 6–7, which were translation validated in Japanese [19–21]. Self-reported physician’s diagnosis was also included as the definition of AD.

### Covariates

The following items were analyzed as potential confounding factors during pregnancy: mother’s age at delivery (<25, 25–29, 30–34, ≥35 years), maternal body mass index (BMI) before pregnancy (<18.5, 18.5–19.9, 20–22.9, 23–24.9, ≥25 kg/m^2^), maternal smoking during pregnancy (yes, no), maternal educational status (<13, 13–15, ≥16 years), maternal allergic history including AD, asthma, hay fever, food allergy, and allergic conjunctivitis (yes, no). Also, the following items were analyzed as potential confounding factors during childhood: child’s sex (male, female), birth weight (<2500, 2500–2999, 3000–3499, ≥3500 g), mode of delivery (vaginal, cesarean section), nutrition until six months of age (exclusive breastfeeding, partial breastfeeding, formula feeding), day care attendance at six months of age (yes, no), and keeping pets indoors (yes, no). We obtained the data regarding maternal BMI (height and weight) from medical records transcription at enrollment; maternal history of allergic diseases and smoking from the questionnaire during the first trimester; maternal educational status from the questionnaire during the second or third trimester; maternal age, child’s sex, child’s birthweight from medical records transcription at delivery; regarding child’s feeding style, day nursery attendance from the questionnaire at six months after birth; and pet ownership from the questionnaire at 1.5 years after birth.

### Statistical analysis

The association between constipation at 1 year of age, and cumulative incidence of AD until 1.5, 2, and 3 years of age was assessed. We used multivariable logistic regression analyses to estimate the crude, and adjusted odds ratios (ORs and aORs, respectively) of the AD with 95% confidence intervals (CI), using the “normal” group as a reference. We also performed the regression analyses when AD was defined solely by the responses to the questionnaire and when AD was defined solely by the self-reported physician’s diagnosis. For multivariate analysis, mother’s age at delivery, maternal BMI before pregnancy, smoking during pregnancy, educational status, allergic history (AD, asthma, hay fever, food allergy, and allergic conjunctivitis), child’s sex, birth weight, mode of delivery, feeding pattern, day care attendance, and keeping pets indoors were adjusted.

We conducted all statistical analyses using SAS statistical software version.9.4 (SAS Institute Inc., Cary, NC, USA). *p* < .05 was considered statistically significant.

## Results

There were 104,062 fetal records in the JECS 2019 dataset, and 62,777 participants met the study inclusion criteria (Fig. 1). Table 1 summarizes the sociodemographic and medical characteristics of the participants. Overall, 14,188 children (22.6%) were suffered from AD until 3 years of age. Table 2 presents ORs and aORs with 95% CI of the association between constipation at 1 year of age, and cumulative incidence of AD until 3 years of age. The cumulative incidence of AD was significantly higher among constipated children who had had ≤2 bowel movements per week at 1 year of age, with aOR of 1.18 (95% CI, 1.01–1.38). Such an association was similarly observed for the cumulative incidence of AD until 2 years of age, and at 1.5 years of age, with aOR of 1.18 (95% CI, 0.99–1.40), and 1.15 (95% CI, 0.92–1.43), respectively.

**Table 1:** Characteristics of the participants

	**Total (%)** **(N = 62,777)**		**Cases (%)** **(n = 14,188)**	
**Frequency of bowel movements at 1 year of age (per week)**				
Almost everyday	54,672	(87.1)	12,328	(86.9)
5–6	2,624	(4.2)	561	(4.2)
3–4	4,631	(7.4)	989	(7.4)
≤2 times per week	850	(1.4)	207	(1.5)

**(Maternal and family characteristics)**				
Maternal age at delivery (year)				
<25	5,205	(8.3)	1,235	(8.7)
25–29	17,090	(27.2)	4,013	(28.3)
30–34	22,835	(36.4)	5,112	(36.0)
≥35	17,647	(28.1)	3,828	(27.0)

Maternal history of atopic dermatitis				
No	53,943	(85.9)	11,441	(80.6)
Yes	8,834	(14.1)	2,747	(19.4)

Maternal history of asthma				
No	56,577	(90.1)	12,419	(87.5)
Yes	6,200	(9.9)	1,769	(12.5)

Maternal history of hay fever				
No	40,780	(65.0)	8,688	(61.2)
Yes	21,997	(35.0)	5,500	(38.8)

Maternal history of food allergy				
No	60,052	(95.7)	13,409	(94.5)
Yes	2,725	(4.3)	779	(5.5)

Maternal history of allergic conjunctivitis				
No	56,836	(90.5)	12,522	(88.3)
Yes	5,941	(9.5)	1,666	(11.7)

Maternal BMI before pregnancy (kg/m^2^)				
<18.5	10,097	(16.1)	2,306	(16.3)
18.5–19.9	15,648	(24.9)	3,472	(24.5)
20–22.9	24,035	(38.3)	5,396	(38.0)
23–24.9	6,646	(10.6)	1,569	(11.1)
≥25	6,314	(10.1)	1,437	(10.1)
Missing	37	(0.1)	8	(0.1)

Maternal smoking during pregnancy				
No	59,718	(95.1)	13,384	(94.3)
Yes	2,352	(3.8)	636	(4.5)
Missing	707	(1.1)	168	(1.2)

Maternal educational status (year)				
≤12	21,136	(33.7)	4,772	(33.6)
13–15	26,822	(42.7)	6,185	(43.6)
≥16	14,175	(22.6)	3,071	(21.7)
Missing	644	(1.0)	160	(1.1)

Child’s sex				
Male	31,234	(49.8)	7,112	(50.1)
Female	31,543	(50.3)	7,076	(49.9)

Birth weight (g)				
<2,500	5,003	(8.0)	1,104	(7.8)
2,500–2,999	24,532	(39.1)	5,526	(39.0)
3,000–3,499	26,342	(42.0)	5,966	(42.1)
≥3,500	6,745	(10.7)	1,558	(11.0)
Missing	155	(0.3)	34	(0.2)

Mode of delivery				
Vaginal delivery	50,935	(81.1)	11,541	(81.3)
Caesarean section	11,571	(18.4)	2,584	(18.1)
Missing	271	(0.4)	63	(0.4)

Nutirtion until the age of 6 months				
Exclusive breastfeeding	24,700	(39.4)	5,683	(40.1)
Partial breastfeeding	24,891	(39.7)	5,579	(39.3)
Formula feeding	12,720	(20.3)	2,826	(19.9)
Missing	466	(0.7)	100	(0.7)

Day care attendance at 6 months of age				
No	58,039	(92.5)	12,903	(90.9)
Yes	4,136	(6.6)	1,140	(8.0)
Missing	602	(1.0)	145	(1.1)

Pet ownership at 1.5 years of age				
No	51,390	(81.9)	11,609	(81.8)
Yes	10,143	(16.2)	2,312	(16.3)
Missing	1,244	(2.0)	267	(1.9)

BMI: body mass index

**Table 2:** Association between frequency of bowel movements and cumulative incidence of atopic dermatitis

	**Frequency of bowel movements**			

	**Alomost everyday**	**5–6 times/wk**	**3–4 times/wk**	**≤2 times/wk**
Cumulative incidence until 3 years of age				
No. at risk	54,672	2,624	4,631	850
No. of cases (%)	12,328 (22.6)	598 (22.8)	1043 (22.5)	219 (25.8)
Crude OR (95% CI)	ref	1.01 (0.92–1.11)	1.00 (0.93–1.07)	1.19 (1.02–1.39)
Multivariable OR (95% CI)^a^	ref	1.02 (0.93–1.12)	1.00 (0.93–1.07)	1.18 (1.01–1.38)

Cumulative incidence until 2 years of age				
No. at risk	56,579	2,717	4,805	882
No. of cases (%)	9,253 (16.4)	468 (17.2)	778 (16.2)	165 (18.7)
Crude OR (95% CI)	ref	1.06 (0.96–1.18)	0.99 (0.91–1.07)	1.18 (0.99–1.40)
Multivariable OR (95% CI)^a^	ref	1.07 (0.97–1.19)	0.99 (0.91–1.07)	1.18 (0.99–1.40)

Cumulative incidence until 1.5 years of age				
No. at risk	57,820	2,782	4,885	905
No. of cases (%)	5,175 (9.0)	263 (9.5)	437 (9.0)	92 (10.2)
Crude OR (95% CI)	ref	1.06 (0.93–1.21)	1.00 (0.90–1.11)	1.15 (0.93–1.43)
Multivariable OR (95% CI)^a^	ref	1.07 (0.94–1.22)	1.00 (0.90–1.11)	1.15 (0.92–1.43)

CI: confidence interval; OR: odds ratio

^a^Adjusted by maternal characteristics such as mother’s age at delivery, maternal body mass index before pregnancy, maternal smoking, maternal educational status, maternal allergic history including atopic dermatitis, asthma, hay fever, food allergy, and allergic conjunctivitis. Also adjusted by child’s characteristics such as child’s sex, birth weight, mode of delivery, exclusive breastfeeding for 6 months or more, day care attendance, and pet ownership.

The above associations were similarly observed when AD was defined solely by the responses to the questionnaire (Supplementary Table 1). However, when AD was defined solely by the physician’s diagnosis, no associations were observed.

## Discussion

To the best of our knowledge, this is the first study to investigate the association between constipation and AD using large cohort data of children. In this study, constipation (the frequency of defecation ≤2 times per week) at 1 year of age was associated with a higher risk of AD until 3 years of age, compared to no constipation. No excess risk of AD was found for the frequency of defecation of 5–6 times per week, or 3–4 times per week.

These association did not change materially when AD was defined solely by the responses of “yes” to the questionnaire. However, no associations were observed when AD was defined solely by the physician’s diagnosis. Many physicians were general pediatricians but not an allergist or a dermatologist so that the diagnosis of AD varied largely which led to the dilution of the associations.

Two previous reports examined the association between constipation, and the development of AD. Tokunaga et al. conducted a cross-sectional study of approximately 20,000 Japanese high school students aged 15 years and older [12], and showed that constipation, based on self-administered questionnaire, increased the prevalence of AD (diagnosed by an allergist, or a dermatologist), by approximately 17%, compared with the normal group. Huang YC et al. conducted a retrospective cohort study using the Taiwan National Insurance database [13], and reported that patients with constipation (ICD-9-CM codes = 564.0: with at least three outpatient visits or one hospitalization) had a higher risk of developing AD (ICD-9-CM codes = 691) than non-constipated patients, with adjusted hazard ratios of 1.89 (95% CI: 1.58–2.27) for ages <6 years, and of 2.31 (95% CI: 2.17–2.46) for all ages including ≥65 years.

Although the pathophysiological mechanisms between constipation and AD have not been fully elucidated, Lee SY et al. proposed that the gut microbiota under no constipation may influence the pathogenesis of host AD through three pathways [22]. The first is the probiotic-mediated immunological pathway, which can lead pro-inflammatory or anti-inflammatory status through the production of various kinds of cytokines, depending on the probiotic strains. The second is the metabolic pathway, in which dietary fiber ingested by the host is metabolized by the gut microbiome to increase the amount of short-chain fatty acids in the intestinal tract, which have anti-inflammatory effects. Moreover, Kynurenic acid produced by the ingestion of certain lactic acid bacteria reduces itching sensation. The third is a neuroendocrine pathway in which tryptophan produced by the gut microbiota aggravates itching sensation, while gamma aminobutyric acid produced by some lactic acid bacteria reduces itching sensation.

There are several limitations of this study. The JECS questionnaire differed from the ROME criteria [17, 18], which is used globally. The prevalence of childhood constipation was reported to be 9.5% in the meta-analysis by Koppen et al. [23] in which constipation was defined by ROME criteria; the present study showed that the proportion of constipated children who had ≤2 bowel movements per week at 1 year of age was only 1.35%, indicating that constipation may have been underestimated. The second is, since the dataset used in this study is mostly Japanese, it is not clear whether the findings of this study can be generalized to other ethnic groups. Finally, residual confounding could have occurred from unmeasured confounding variables, such as history of post-natal antimicrobial use.

Our study provides a new insight for the role of constipation on the development of AD in early childhood albeit the magnitude of excess risk was small and clinical significance may be limited. It is possible to detect AD at an early stage by recognizing the signs of constipation using the frequency of defecation, and the early treatment of constipation could prevent the risk or worsening of AD, which will need to be verified by further investigation.

## Conclusion

Functional constipation at 1 year of age was associated with a slightly higher risk of AD until 3 years of age. Further studies are necessary to examine the association after 3 years of age because AD may develop at later ages.

## Abbreviations

AD: atopic dermatitis
aOR: adjusted odds ratio
BMI: body mass index
CI: confidence intervals
ICD-9-CM: International Classification of Diseases 9th Revision Clinical Modification
ISAAC: International Study of Asthma and Allergies in Childhood
JECS: Japan Environment and Children’s Study
OR: odds ratio

## Supplementary information

**Additional file 1:** **Supplemental Table 1** Association between frequency of bowel movements and cumulative incidence of atopic dermatitis.

## Declarations

### Availability of data and material

Data are unsuitable for public deposition due to ethical restrictions and legal framework of Japan. It is prohibited by the Act on the Protection of Personal Information (Act No. 57 of 30 May 2003, amendment on 9 September 2015) to publicly deposit the data containing personal information. Ethical Guidelines for Medical and Health Research Involving Human Subjects enforced by the Japan Ministry of Education, Culture, Sports, Science and Technology and the Ministry of Health, Labour and Welfare also restricts the open sharing of the epidemiologic data. All inquiries about access to data should be sent to: jecs-en@nies.go.jp. The person responsible for handling enquiries sent to this e-mail address is Dr Shoji F. Nakayama, JECS Programme Office, National Institute for Environmental Studies.

### Author’s information

ORCID ID: Yoshihiko Takano https://orcid.org/0000-0002-7105-8328
